# The Impact of the COVID-19 Pandemic on Respiratory Illness Admissions at a Single Academic Institution in Arkansas

**DOI:** 10.3390/ijerph191912533

**Published:** 2022-10-01

**Authors:** Mallory Heft, Joshua Mueller, Hanna Jensen, Nicholas Kaukis, Mollie Meek

**Affiliations:** 1 College of Medicine, University of Arkansas for Medical Sciences, Little Rock, AR 72205, USA; 2 Department of Internal Medicine, University of Arkansas for Medical Sciences, Little Rock, AR 72205, USA; 3 Department of Radiology, University of Arkansas for Medical Sciences, Little Rock, AR 72205, USA; 4 Department of Biostatistics, University of Arkansas for Medical Sciences, Little Rock, AR 72205, USA

**Keywords:** public health, rural health, COVID-19

## Abstract

Background: The first reported COVID-19 case in Arkansas was on 11 March 2020, two months after the first reported case in the United States. We sought to analyze rates of respiratory illness and influenza tests during the 2019/2020 influenza season compared to pre-pandemic years to assess whether there were higher rates of respiratory illness than expected, which may suggest undiagnosed COVID-19 cases. Methods: Using data collected from the data warehouse of the largest hospital in Arkansas, ICD-9 and ICD-10 codes related to respiratory illness were identified for 1 October to 1 May 2017–2020. Results: We identified 25,747 patients admitted with respiratory illness during the study. We found no significant difference in the rate of monthly admissions with respiratory illness between seasons (*p* = 0.14). We saw a significant increase in the number of influenza tests ordered in 2019/2020 (*p* < 0.01). Conclusions: The rate of hospitalizations with respiratory illness did not significantly increase during the 2019/2020 season; however, influenza testing increased without a statistically significant difference in positivity rate. The increase in ordered influenza tests indicates an increased clinical suspicion, which may suggest a rise in pre-hospital viral illness associated with COVID-19.

## 1. Introduction

The first COVID-19 case reported in Arkansas was on 11 March 2020, almost two months after the first reported case in the United States (US) [1]. The largest hospital in Arkansas, the University of Arkansas for Medical Sciences (UAMS), admitted its first patient with COVID-19 on 13 March 2020 [1]. Most research studies at the beginning of the pandemic focused on larger and more urbanized states, such as California, New York, and Washington. Testing for SARS-CoV-2 was more robust in these states than in rural states [2]. This may be the result of deficits in testing capabilities due to fewer authorized labs or limited supplies and storage abilities.

From the beginning of the pandemic, it was quickly discovered that COVID-19 was disproportionately affecting individuals with diabetes mellitus, hypertension, and obesity [3]. According to the Centers for Disease Control and Prevention (CDC), Arkansas is among the highest prevalence states for diabetes mellitus, hypertension, and obesity [4,5,6]. Despite Arkansas having a largely vulnerable population, there was a delay in identified emergence of COVID-19 in our state compared to more urbanized states. While many rural states experienced their first COVID-19 cases later than more urbanized areas, it is unclear whether it was an actual delay in emergence or a false sense of security due to decreased research and testing.

This study sought to find if there was a difference in the rate of respiratory illness admissions at UAMS during the 2019/2020 influenza season compared to the previous seasons beginning in 2017. A difference in respiratory illness admissions could imply an earlier effect of COVID-19 than detected by testing. Furthermore, we analyzed the positivity rates of influenza tests performed. Increases in respiratory illness without a parallel increase in positivity rates of common respiratory illnesses may suggest COVID-19 cases that were undetected.

## 2. Methods

### 2.1. Data Collection

An institutional review board (IRB) exemption was granted for this retrospective study of data from the in-house data warehouse, Arkansas Clinical Data Repository (AR-CDR), which stores information from the electronic medical records for patients from the UAMS in Little Rock, AR. The data extracted from the AR-CDR included diagnostic information, procedural information, influenza volume of tests, and influenza test results. We used diagnostic information to identify the volume of patients with specific comorbidities of diabetes, hypertension, and obesity. Our study included any person admitted with an ICD-9 or ICD-10 code corresponding to respiratory illness (Table 1) for influenza seasons between 2017–2020, with 1 October to 1 May representing each season. ICD-9 and ICD-10 codes were chosen based on a literature review concerning previous influenza surveillance studies to determine which codes would help extract the appropriate data [7,8,9,10,11,12,13]. Patients younger than 15 were excluded due to studies indicating COVID-19 was not primarily affecting this age group at the beginning of the pandemic [3,14,15]. The original dataset given to the principal investigator included only patients admitted to UAMS during the specific time frame with the ICD-9 or ICD-10 codes corresponding to respiratory illness. Each patient had a unique de-identified code.

### 2.2. Statistical Analysis

To ensure comparability across seasons, we calculated weekly and monthly rates of respiratory-related admissions, volume of influenza tests, and positivity rate of influenza tests, each standardized by the number of admitted patients within a given week. Single-factor ANOVA was conducted to evaluate the difference in rates of respiratory-related illness admissions across seasons. Chi-squared tests were utilized to assess volume counts for influenza tests and comorbidities. The statistical software used was R version 4.1.0, and statistical significance was assumed at (*p* < 0.05).

## 3. Results

During 1 October 2017–2020 through 1 May seasons, 25,747 patients were admitted for respiratory illness at UAMS in Little Rock, AR. Total yearly admission volumes for respiratory illnesses increased from 2017/2018 to 2018/2019; however, they minimally changed from 2018/2019 to 2019/2020 (Table 2). When comparing the rate of monthly admissions for respiratory illness across seasons, there was no significant difference (*p* = 0.14). Respiratory admissions appear to peak in December and January (Table 2). There was a slightly higher admission rate for 2019/2020 from October through February compared to other seasons (Table 2); however, there is no statistically significant difference between these rates (*p* = 0.32).

Despite no significant difference in the rate of positive influenza tests between seasons, there was a significant difference in the volume of influenza tests ordered (*p* < 0.01). As shown in Table 3, more influenza tests were performed in the 2019/2020 season without a statistically significant change in positivity rate (*p* = 0.51).

The two most common comorbidities were diabetes mellitus and hypertension. A statistically significant increase in patients having diabetes mellitus (DM), hypertension (HTN), and obesity was found in the 2019/2020 season (DM *p* < 0.01, HTN *p* < 0.01, and Obesity *p* < 0.01).

## 4. Discussion

This study showed no difference in the rate of hospital admissions with respiratory illness at a single academic institution for the 2019/2020 season. However, data shows a statistically significant increase in influenza tests ordered in the 2019/2020 season. There was no significant difference in the positivity rate for influenza across seasons. There was a statistically significant increase in patients with diabetes, hypertension, and obesity in 2019/2020 compared to the previous years.

We hypothesized that the admission rate for patients with respiratory illness at UAMS would be higher for the 2019/2020 season, but with less frequently positive influenza tests, potentially due to the unknown presence of COVID-19, leading to misdiagnosis as influenza or other respiratory illness. While some believe living in a rural community insulates and protects individuals from infection compared to urban areas, this may not be the case. Rural jobs tend to be industrial and, therefore, cannot be moved online [16]. This requires showing up and working in close quarters with others. Henning-Smith et al. reported that rural areas, on average, have older populations and more people with underlying health conditions [17].

Despite Little Rock, AR, not being a rural area, UAMS serves as the state’s primary tertiary care medical center. According to the University of Arkansas Department of Agriculture, 44% of Arkansas residents live in rural areas, while only 14% of the US population lives in rural areas [18]. These factors make social distancing more difficult and increase the risk of COVID-19 infection. Huang et al. published a study concerning the epidemiology of the COVID-19 pandemic in South Carolina from 1 March to 5 September 2020. They found that when comparing case rates of COVID-19 between urban versus rural counties in South Carolina, rural counties had higher case rates [19]. According to the South Carolina Institute of Medicine and Public Health, 29% of the state’s population lives in rural areas [20]. This highlights the importance of increasing knowledge concerning the epidemiology of the COVID-19 pandemic in rural states.

The clinical presentation of COVID-19 is nonspecific, like other respiratory illnesses, including influenza. Retrospective studies from the pandemic’s beginning in Wuhan, China, reported that 94% of patients with COVID-19 presented with fever, and 72–82% presented with cough [14,15]. Data released from the US CDC also reported fever and cough as the most prominent symptoms of COVID-19 during the early pandemic [3]. Previous studies conducted in the US to evaluate the clinical presentation of influenza have found fever and cough to be the strongest indicators of influenza [11,21,22]. With similar clinical presentation and limitations in diagnostic tests, it is possible to misdiagnose COVID-19 as influenza, especially in the months prior to March 2020, when we were unaware of its presence in Arkansas, yet it was in the US.

Due to decreased testing rates and less stringent restrictions on travel and physical distancing in Arkansas and other rural states, we wanted to see if there was an increase in inpatient admissions for respiratory illness potentially due to undiagnosed cases of COVID-19. The volume of influenza tests ordered significantly increased during the 2019/2020 season, suggesting a higher clinical suspicion for influenza or respiratory illness symptoms, leading to increased testing. The positivity rate tests for influenza showed no statistically significant change across seasons. Furthermore, the weekly influenza report released on 7 March 2020 from the Arkansas Department of Health indicates that the percent of outpatient visits with influenza-like illness was higher for 2019/2020 compared to 2018/2019 and higher than the average for 2013–2018 [23]. Likewise, the same report shows a sharp decrease in positive influenza tests for influenza A and B around week 6 of 2020 [23]. Considering COVID-19 has a similar presentation to influenza, it would not be unreasonable to postulate these people with negative influenza tests could have been infected with COVID-19. Our data show a statistically significant increase in patients admitted for respiratory illness with comorbidities of hypertension, obesity, and diabetes mellitus for the 2019/2020 season, which corresponds to a population known to be more vulnerable to COVID-19. 

Interestingly, the SHARE study recently published by Veronese et al. found individuals older than 50 who received the influenza vaccine were at lower risk of COVID-19 infection, symptomatic forms of COVID-19, and hospitalization for COVID-19 [24]. According to the CDC in Arkansas, during 2019/2020, there was an estimated influenza vaccine coverage rate of 53.6% [25]. Though future research will need to be conducted to elucidate this relationship further, this could potentially explain why there was not an increase in hospital admissions for respiratory illness in 2019/2020, which represents more severe forms of COVID-19.

A limitation of this study is that ICD-9 and ICD-10 codes used in the study were selected in June 2020. At the time, little was known about COVID-19 surveillance; therefore, this study was modeled like other influenza surveillance studies, which could have led to selection bias. Another limitation of this study was that the original dataset had incomplete data for patient characteristics such as age and sex; thus, we could not analyze those characteristics in this study.

## 5. Conclusions

Various factors make Arkansas residents a more vulnerable population to COVID-19 infection, yet our data suggest a delayed impact in the influx of COVID-19 in Arkansas. Despite no difference in respiratory illness admissions at UAMS for the 2019/2020 season, the increased influenza testing in the same season suggests an increased clinical suspicion of respiratory illness. This finding, in addition to data provided by the Arkansas Department of Health for the 2019/2020 influenza season, could potentially correlate to a subtle increase in community COVID-19 cases. Considering our population only included hospitalized patients, it could be postulated that respiratory illness was increasing in outpatient clinics throughout Arkansas prior to seeing the influx of more severe cases in the hospital setting. Future studies could compare the 2020/2021 season to previous years to see if respiratory illness admissions increased at UAMS, considering the state did not truly experience the first peak wave of COVID-19 cases until January 2021 [26].

## Figures and Tables

**Table 1:** ICD-9 Or ICD-10 Codes Utilized to Extract Data from the Institutional Data Warehouse at the University of Arkansas for Medical Sciences.

Diagnosis	ICD-9 Code	ICD-10 Code
Influenza	487–488	J09–J11
Pneumonia	480–486	J12–J18
Acute Upper Respiratory Infection	460–465	J00–J06
Respiratory Diseases	466–519	J00–J99
Circulatory Diseases	390–459	I00–I99
Fever, Unspecified	780.60	R50.9
Cough	786.20	R05
Throat Pain	786.20	R07
Unspecified Viral Infection	079.99	B97.89
Unspecified Otitis Media	382.90	H66.9
Respiratory Arrest	799.1	R09.2
Shortness of Breath	786.05	R06.02
COVID-19, Confirmed Diagnosis	-	U07.1
Symptoms of COVID-19 with known exposure	-	Z20.828
Coronavirus infection, unspecified	-	B34.2
Coronavirus as cause of diseases classified elsewhere	-	B97.29

**Table 2:** Total Yearly Volume and Monthly Rate * of Patients Admitted to UAMS with an ICD-9 or ICD-10 code Correlated to Respiratory Illness.

Season (1 October–1 May)	Volume of Respiratory Illness Admissions	October	November	December	January	February	March	April
2017–2018	8313	11.7%	13.2%	14.1%	14.6%	13.8%	15.3%	14.6%
2018–2019	8719	12.4%	13.3%	14.4%	14.4%	13.6%	15.3%	14.2%
2019–020	8715	12.9%	14.8%	15.0%	15.8%	15.4%	14.2%	9.6%

* The monthly rate represents patients admitted with ICD-9 or ICD-10 codes corresponding to respiratory illness out of the total volume of patients admitted to UAMS each month.

**Table 3:** Total Volume of Influenza Tests Performed with the Corresponding Positivity Rate.

Season (1 October–1 May)	Total Number of Influenza Tests Performed	Influenza Positive Test Rate
2017–2018	2594	3.2%
2018–2019	3492	1.8%
2019–2020	4273	2.0%

## Data Availability

All corresponding data can be accessed at this google drive link: https://drive.google.com/drive/folders/1IVovA8J2fPF4rs5qHTQ8vdWNENSJLImG?usp=sharins (accessed on 30 August 2022).
